# Human Immunodeficiency Virus and Trans Women: A Literature Review

**DOI:** 10.1089/trgh.2018.0005

**Published:** 2018-12-28

**Authors:** Rusi Jaspal, Lauren Kennedy, Shema Tariq

**Affiliations:** ^1^ Faculty of Health & Life Sciences, De Montfort University, Leicester, United Kingdom.; ^2^ Minority Research Profile, Åbo Akademi University, Turku, Finland.; ^3^ Centre for Clinical Research in Infection and Sexual Health, University College London, London, United Kingdom.

**Keywords:** HIV, risk, syndemic, transgender

## Abstract

Trans women are a key, yet under-researched, population in the HIV epidemic. However, there remains a paucity of data on the health and wellbeing of trans women at risk of, or living with, HIV in the United Kingdom. This article provides a narrative review of key empirical research into HIV among trans women. In an effort to explore individual and social factors in relation to HIV in this population, we outline key tenets of identity process theory from social psychology and the concept of structural violence from medical anthropology. We focus on published studies around the following themes: (1) epidemiological data, (2) syndemic factors (3) barriers to social support, (4) HIV and gender transitioning, and (5) access to and engagement with health care. We identify lacunae and thus call for United Kingdom-based research in the following areas: (1) the prevalence and incidence of HIV in trans women, (2) the impact of syndemic factors on HIV risk and acquisition in trans women, (3) the nature of social support for coping with syndemic factors, (4) the interface of gender transitioning and HIV, and (5) barriers to accessing HIV prevention and care services. There is great scope (and urgency) for research into HIV among trans women, especially in the United Kingdom, to reduce incidence in this group, to enhance engagement in HIV care across the care continuum, and to improve the health and wellbeing of those living with HIV. A tentative model for HIV prevention and care is presented in this article.

## Introduction

Almost 40 million people are living with HIV globally.^1^ Advances in treatment mean that, for those who have access to HIV medication (known as antiretroviral therapy [ART]), life expectancy now approaches that of the general population.^2^ A key aspect of the management of HIV is the HIV treatment cascade (or HIV care continuum).^3^ This model of care outlines the steps that people living with HIV must take from initially being tested and diagnosed, to being established on effective ART and achieving viral suppression (having a very low level of virus in the body, which drives the vastly improved clinical outcomes in HIV and reduces horizontal transmission of HIV).

The Joint United Nations Program on HIV/AIDS (UNAIDS) have set a target of 90-90-90; that is, by 2020, for 90% of all people living with HIV to know their status, for 90% of those diagnosed with HIV to have sustained ART, and for 90% of those on ART to have achieved viral suppression.^4^ However, progress toward these goals is not equitable, with certain groups not reaching these targets.^5^ These key populations are at higher risk of acquiring HIV, and may also face significant challenges in engaging in HIV care. This in turn may lead to poorer clinical outcomes for the patient and to increased risk of onward HIV transmission.

Trans women are a key population in the prevention and management of HIV. However, there remains a paucity of data on the health and wellbeing of trans women at risk of, or living with, HIV. Much HIV research focuses on men who have sex with men (MSM), some of which also include trans women in the participant sample.^6,7^ There has been a tendency in the literature to conflate MSM with trans women, and often the data are not disaggregated. This tendency not only obscures differences in epidemiology, access to health care, and lived experiences between these groups, but it also fundamentally fails to recognize trans women's identities and their specific health care needs.

This article provides a narrative review of key empirical research into HIV among trans women. The aim is not to provide a systematic review of the literature but rather to outline and discuss overarching research themes in the current literature on HIV among trans women. In an effort to promote the analysis of both individual and social factors in relation to HIV in this population, we advocate the use of identity process theory^8^ from social psychology and the concept of structural violence^9^ from medical anthropology. In this review, we focus on the following themes: (1) epidemiological data, (2) syndemic factors, (3) barriers to social support, (4) HIV and gender transitioning, and (5) access to, and engagement with, health care. We conclude by describing a tentative model for preventing HIV and increasing access to HIV care among trans women, and by presenting recommendations for future research into HIV among trans women.

## Theoretical Frameworks

When considering HIV among trans women, we propose the use of theoretical perspectives from both psychology and critical medical anthropology, in an effort to bridge the individual and the structural levels of analysis. Trans women living with HIV face a multifaceted social stigma, as a result of both gender identity and HIV status.^10^ It is important to understand how trans women react to, and cope with, social stigma, as exposure to stigma can challenge one's sense of self and induce negative affect, with adverse outcomes for both physical and psychological wellbeing.^11^ A theoretical approach that captures multiple levels of analysis is crucial.

### Identity process theory

**FIG. 1.**
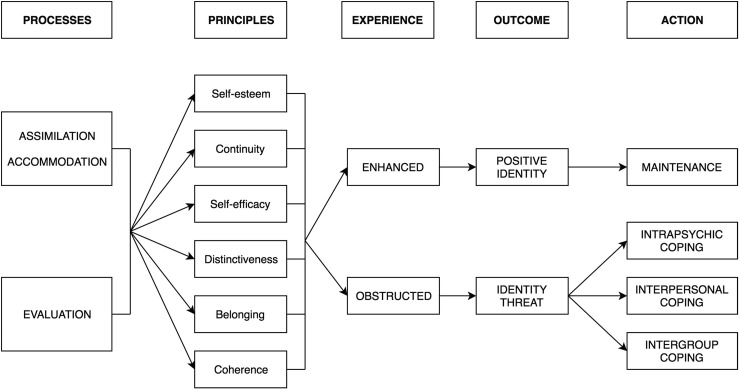
Identity process theory (from Jaspal^32^).

Identity process theory^8^ from social psychology can offer fruitful insights into the ways in which trans women at risk of, or living with, HIV construct, manage and protect identity in the face of social stigma. As demonstrated in Figure 1, the theory postulates that individuals construct their identity through engagement in two processes: assimilation-accommodation, and evaluation. For instance, a trans person diagnosed with HIV will incorporate their new HIV status into their existing identity structure and make room for it within this structure, possibly by rethinking other aspects of their identity, such as their ability or desire to undergo gender reassignment.

The identity processes are in turn guided by various motivational principles, namely self-esteem; self-efficacy; distinctiveness; continuity; and coherence. When these principles are compromised, for instance, by changes in one's social context, identity is said to be threatened and the individual will deploy strategies for coping with the ensuing threats. For instance, the experience of transphobia could plausibly challenge self-esteem among trans people.^12^

Some strategies to cope with threat function at a psychological level (e.g., denial), whereas others are enacted at interpersonal and intergroup levels (e.g., isolation, deriving social support). Some strategies are proactive while others are maladaptive. However, all of the strategies aim to maintain or restore high levels of the identity principles. Thus, by drawing on tenets of identity process theory, it may be possible to understand particular patterns of thought and action that could increase the risk of HIV infection or the risk of poor engagement with HIV care, respectively.

### Structural violence

Sociostructural approaches can complement the individualized analysis of thought and behavior offered by identity process theory. Structural violence, as conceptualized by Galtung, refers to violence that extends beyond the physical domain and that is “present when human beings are being influenced so that their actual somatic and mental realizations are below their potential realizations.”^9^ Structural violence is not perpetrated by a subject but is instead “built into the structure and shows up as unequal power and consequently unequal life chances” as a result of the uneven distribution of resources and power, and is often not perceived directly.

Structural violence is a central organizing principle in Critical Medical Anthropology (the branch of medical anthropology that uses critical theory and an explicit consideration of issues of power to explore social inequality and ill health), with writers such as Bourgois^13^ and Scheper-Hughes^14^ focusing on how the macro-level forces of poverty, racism, gender inequality, and political violence come to be embodied by people in the form of ill health.

Paul Farmer in his work in Haiti, Russia, Rwanda, and the United States has challenged the notion of individual risk behaviors, showing how increased vulnerability to HIV and poor access to HIV care are predominantly driven by poverty, lack of health infrastructure, unequal gender relations, discriminatory practices, and aspects of national and international policy.^15^ There is, therefore, a clear precedent for employing the concept of structural violence when considering disparities in health, especially in work on HIV and marginalized groups, such as trans women. The principle of structural violence sheds light on the social context in which psychological stressors can occur and adversely impact physical health outcomes among trans women, a group with decreased social capital, as outlined in this narrative review.

## Methods

L.K. undertook a systematic search of the following electronic library databases in July–August 2017: PubMed and PsycINFO. Our aim was to identify original research articles that examined HIV in trans women, with a focus on HIV epidemiology, pre-exposure prophylaxis (PrEP), risk factors for HIV acquisition, access to HIV care, and HIV clinical outcomes.

The search strategy was developed by L.K. and R.J. The search was not restricted to a particular time period, although we endeavor to include the most recent data wherever possible. Search terms included: “transgender”, “transsexual” (to capture older literature), and “women”, all in combination with the terms “HIV” or “AIDS”. We applied these search terms to titles in all databases, and restricted all searches to studies in adults only. We only included studies published in English. We did not include gray literature.

We selected studies where at least one of the study populations was trans women; all study designs were eligible. In addition, bibliographies of review articles were crossreferenced and we handsearched available abstracts from the following conferences: British HIV Association, Conference for Retroviruses and Opportunistic Infections, and the International AIDS Society. Final selection was discussed by all authors until consensus was reached.

In this review, we employ tenets of identity process theory and structural violence to develop tentative hypotheses about HIV risk, and enhancing HIV prevention and care. This is intended to contribute to the development of a systematic and theoretically informed research program in this key population.

## Epidemiological Data

A 2013 meta-analysis of data from 39 studies in 15 countries, including 11,066 transgender women revealed an HIV prevalence of nearly 20% among trans women globally, with prevalence varying from 18% in low- and middle-income countries, to 22% in high-income countries.^16^ The authors report that, based on these studies, trans women are 49 times more likely than other adults to be living with HIV. However, these data must be considered with caution given that trans women are also less likely to be engaged in health care, which may mean that a significant number of people in this population are living with undiagnosed HIV.^17^

Indeed, a systematic review has indicated a discrepancy between rates of self-reported HIV prevalence among trans women in the United States, and laboratory-confirmed HIV infection, suggesting that many trans women living with HIV may not be aware of their HIV status and are therefore unable to avail themselves of life-sustaining ART.^18^ Furthermore, it is suggested that trans women are at higher risk of HIV acquisition than other transgender groups, such as transgender men and nonbinary transgender people,^19^ although transgender MSM are also thought to be at a high risk.^20^

It is important, however, to note that there are other intersecting sociodemographic disparities in relation to HIV vulnerability that may also affect trans women, such as minority ethnic status and socioeconomic deprivation.^21^ For instance, a United States study of adolescent trans women has found that African American participants were significantly more likely to have acquired HIV compared with their White counterparts.^22^ We know from existing research that the HIV epidemic among trans women is also highly patterned by geographical region.^16,23^ Therefore, it is not easy to extrapolate findings from global data to the United Kingdom context.

Until relatively recently, there has been an absence of data on HIV in trans women in the United Kingdom. However, in 2017 Public Health England presented some of the first epidemiological data on HIV among trans women. Drawing on the HIV and AIDS Reporting System, which includes data from outpatient HIV services, Delpech et al.^24^ of Public Health England reported that 199 trans individuals accessed HIV care services in 2016 (0.3% of the total patient cohort), and that trans patients were twice as likely as other patients to be diagnosed late (with a CD4 count of <350 mm^3^). However, there are currently no data on either prevalence or incidence of HIV among trans women in the United Kingdom. This can, in part, be attributed to the fact that 2011 United Kingdom Census did not capture the trans and nonbinary (not identifying as only male or only female, or identifying as both) population size in the United Kingdom, although this may change in the 2021 Census.

## Syndemic Factors and HIV Risk

Trans women face structural violence in the form of a multitude of social stressors and structural inequalities, which can plausibly threaten the identity principles of self-esteem, self-efficacy, continuity, and so on and, thus, undermine psychological wellbeing.^7,25–29^

Several studies demonstrate that HIV risk behaviors, such as condomless anal intercourse, sex work, and substance use, may sometimes function as a way of coping (often with unintended negative consequences) with stigma and other social stressors, which we discuss below.^19,27,30,31^ For example, workplace discrimination may lead to workplace exclusion and potentially to engagement in sex work,^32^ and social rejection can lead to substance use as a means of escapism.^33^ It is easy to see how such prejudice and discrimination can undermine the self-esteem and continuity principles of identity.

The co-occurrence and interaction of potentially harmful psychosocial factors and health conditions in a group is often referred to as a syndemic and is well recognized in HIV research.^34^ A syndemic can also be understood in terms of an event or situation, which adversely impacts the identity principles of self-esteem, continuity, self-efficacy, and so on. Understanding these syndemic factors can enable us to identify the opportunities and limitations in relation to both HIV prevention and strategies for engaging trans women living with HIV throughout the HIV care continuum. This can also enable us to understand patterns of cognition and behaviors in a population with an elevated risk of HIV infection.

### Stigma and discrimination

Systemic, institutional and interpersonal discrimination against trans women can lead to negative psychosocial and behavioral outcomes.^28^ The sites of such discrimination are multifarious and can include inter alia the family,^33^ the workplace,^28^ health care providers,^35^ and the criminal justice system,^28^ with trans women often facing multiple and intersecting forces of discrimination and exclusion.

In addition to overt discrimination, such as name-calling and physical violence, trans women may also face more subtle forms of discrimination. For instance, it has been observed that trans gender identity may be erroneously conflated with sexual minority identity (including, as discussed earlier, in research), which can be stigmatizing and distressing.^36^ There is growing evidence that social stigmatization (on the basis of various social categories, such as race, sexuality, gender) predicts engagement in HIV risk behaviors in trans women,^37^ but also in other populations.^31^ These risk behaviors may constitute an attempt to deflect threats to self-esteem associated with social stigma.^38^

### Violence

Several empirical studies exhibit a high prevalence of emotional, physical, and sexual abuse in trans women. In their longitudinal study of trans women, Nuttbrock et al.^30^ note a 29–47% prevalence of psychological and physical gender-related abuse, while Miller^7^ found that trans sex workers were more likely than male sex workers to be physically abused. Several studies exhibit a high prevalence of intimate partner violence perpetrated against trans individuals.^39,40^ Langenderfer-Magruder et al.^39^ found that 31.1% of trans people and 20.4% of cisgender people (people whose gender identity is the same as the sex they were assigned at birth) in their study had ever experienced intimate partner violence.

Furthermore, qualitative data demonstrate the significant burden of physical violence from the clients of trans sex workers.^23^ In their study of trans women with a history of sex work, Nemoto et al.^21^ found that almost 40% of respondents had experienced childhood sexual abuse, which is known to constitute a significant predictor of increased HIV risk.^41,42^ In their study of 60 trans people in Scotland, almost 50% of whom were trans women, Roch et al.^43^ found that 80% of their participants had experienced emotionally, physically, or sexually abusive behavior from an intimate partner. Additionally, data from self-report surveys and needs assessments, hotline call, social service records, and police reports in the United States suggest that trans people are at high risk of multiple types of violence, which begins early in life and continues into adulthood, and that the risk of sexual violence is particularly pronounced.^44^

The experience of abuse can jeopardize the self-efficacy principle of identity as it deprives the individual of feelings of control and competence. All of these forms of abuse are associated with increased HIV risk and poor engagement with HIV care,^31,45^ which can be considered an ineffective coping response to identity threat. Indeed, Poteat et al.^37^ have highlighted the effect of violence and abuse on both HIV risk behaviors and self-reported HIV prevalence among trans women.

### Socioeconomic inequalities

Data from the National Transgender Discrimination Survey^46^ suggest that trans individuals are four times more likely than cisgender individuals to have a household income of <10,000 US Dollars per year. They are at elevated risk of homelessness,^26^ of living in poverty,^47^ and of experiencing difficulties in obtaining and maintaining employment.^35^

This socioeconomic hardship among trans people is also observable in the United Kingdom. For instance, a 2008 survey of lesbian, gay, bisexual, and trans people in Southern England found that only a quarter of trans respondents were in full-time employment, and were three times more likely than non-trans respondents to have an income of <10,000 UK Pounds (∼13,000 US Dollars) a year.^48^ Data from the more recent Stigma Survey have revealed that a third of trans people living with HIV in the United Kingdom (the majority, trans women) report food insecurity and that a quarter had struggled to keep up with financial commitments, although these rates were not dissimilar to the overall population living with HIV.^29^

Socioeconomic inequalities can also challenge identity processes through the potential threats to self-efficacy due to the inability to achieve one's goals and to self-esteem due to the social stigma that is itself associated with poverty.^49^ It is noteworthy that both poverty and homelessness are independently associated with HIV risk behaviors, such as sex work, substance misuse, and condomless sex.^41,50^

### Sex work

Socioeconomic inequalities and the need to fund transition, at least in part, contribute to engagement in sex work, a risk factor for HIV acquisition. There is a higher prevalence of history of sex work in trans women than the general population.^51^ A United States meta-analysis suggested that 24–75% of trans women have engaged in sex work,^52^ whereas a survey study of trans women in Lima, Peru revealed a 63.9% prevalence of sex work in the participant sample.^53^

A meta-analysis of 25 studies has reported that trans women who are sex workers are four times more likely to be HIV positive than cisgender female sex workers, although there was no significant difference in HIV prevalence between sex worker trans women and either non-sex worker trans women or MSM sex workers.^54^ This is significant given that, in a recent report, National AIDS Trust^55^ has estimated a higher prevalence of HIV in trans sex workers than in cisgender sex workers.

### Substance misuse

There is a higher prevalence of substance misuse in trans women than the general population, which has been shown to predict engagement in HIV risk behaviors in this population.^51,56^ It has been speculated that substance misuse may be related to the high prevalence of sex work in trans women, on the one hand, and the desire for escapism from pre-existing mental health issues, on the other.^7,56^ In addition to predicting HIV acquisition, substance misuse is also associated with increased risk of onward HIV transmission because substance misuse in people living with HIV is known to impact adherence to ART, thereby compromising virological control.^57^

In recent years, the practice of “chemsex” (drug use in sexualized settings) has been shown to predict engagement in HIV risk behaviors, such as condomless sex with multiple partners in a group setting, among MSM.^31^ However, there are no data on the prevalence of “chemsex” in trans women. On the whole, substance misuse can be conceptualized in terms of a maladaptive strategy for coping with psychological adversity, such as identity threat. Yet, the practice itself may also be related to threats to identity due to the stigma associated with substance misuse (self-esteem) and to the potential adverse impact for everyday functioning, which can constrain one's competence and control (self-efficacy).

### Mental health

Many of the syndemic factors outlined above have an impact on mental health outcomes. Unresolved identity threat can result in poor mental health outcomes, such as depression, anxiety, depersonalization, and others.^58^ Furthermore, some coping strategies can themselves undermine mental health outcomes. For instance, substance misuse in response to psychological adversity can increase the risk of psychosis.^59^ There is evidence that poor mental health is associated with an increased risk of HIV acquisition and of onward transmission.^60,61^

In a 2012 survey of mental health among trans people in the United Kingdom,^26^ the majority of respondents reported current or past depression, stress, or anxiety; 54% were categorized as having major or mild depression using the Center for Epidemiological Studies-Depression scale. The prevalence of deliberate self-harm among trans people is high, with a prevalence of suicide attempts of between 32% and 52% globally, and 63% of those surveyed in the United Kingdom reported suicidal ideation.^62^ It is noteworthy that there is a positive correlation between structural stigma and suicide attempts.^63^ However, there is no empirical research into mental health outcomes and HIV risk in trans women in the United Kingdom.

## Social Support

Social support is a robust empirical predictor of physical and mental wellbeing^64^ and for coping effectively with psychological adversity and identity threat.^31^ Given the social stigma appended to sexual minorities and trans people, trans women may experience a sense of disconnection from social ingroups and, thus, decreased access to social support. Forbes et al.^19^ have found that social support can enhance resilience in trans women. Consequently, a lack of social support can constrain trans women's capacity to cope with adversity and negatively impact mental and physical health.^65,66^

Westbrook and Schilt^67^ have shown that trans people may have limited access to potentially helpful social spaces, such as HIV support groups, in part because of the stigma surrounding trans gender identity. HIV-related stigma can adversely impact access to social support among trans women living with HIV as a result of the desire to avoid sharing one's HIV status to protect oneself from stigma and rejection.^68^

The ability to draw upon social support to cope with an HIV diagnosis depends on the individual's willingness to share their HIV status with others. In their national study of adults attending for HIV care at a United Kingdom clinic, Daskalopoulou et al.^69^ found that participants who had not told anyone about their HIV status were more likely to report low social support, symptoms of depression and anxiety, and nonadherence to ART, and to have uncontrolled HIV. Yet, sharing one's HIV status is possible only if an individual has a trusted other, which, given the low levels of social capital among trans women outlined above, may preclude this process.

While sharing one's HIV status with others constitutes a positive coping strategy and can provide a multitude of social and psychological benefits to people living with HIV, there are also associated risks. Qualitative research has revealed that trans women living with HIV may feel that an HIV diagnosis could reinforce stigmatizing stereotypes of trans women as “dirty”, preventing them from sharing their diagnosis with others.^56^ Furthermore, the association between an HIV diagnosis (and others knowing about this) and gender-based violence is well recognized among cisgender women living with HIV.^70^ UNAIDS^45^ has identified the high risk of exposure to sexual violence among trans women. There is currently no empirical research into the role, or even availability, of social support in preventing HIV among trans women in the United Kingdom.

## HIV and Gender Transitioning

Gender transitioning is the process whereby trans people undergo change to live in a way that is congruent with their gender identity. Transitioning can occur within a number of spheres, which may be independent of one other—for example, social, legal, and medical. It can potentially challenge the continuity principle of identity in view of the inevitable changes in interpersonal relations that occur during this process. Conversely, barriers to transitioning can be challenging for continuity because of the consequential discrepancy between individuals' desired and actual identities.^71^

Medical transitioning involves physical changes to the body through hormone therapy and/or surgical intervention. Some ART regimens are known to interact with estrogen (one of the main hormones administered for medical transition), thereby reducing exposure to estrogen.^72^ This can adversely impact medical transitioning and induce concerns in trans patients living with HIV regarding the quality of their medical transition. Indeed, a survey of 87 trans women living with HIV in the United States reports high levels of ART nonadherence as a result of concerns about the potential of drug interactions to undermine the transition process,^73^ which has previously been reported by other researchers.^56,63^

This is particularly concerning given that Wilson et al.^74^ found that nonuse of transition-related medical care among trans women (in particular, hormone therapy and breast augmentation) was associated with substance misuse, alcohol misuse, and suicidal ideation. As indicated above, all of these practices are associated with increased risk of HIV acquisition/transmission. It is therefore necessary to understand perceptions of, and attitudes toward, both ART and hormone replacement therapy among trans women living with HIV so as not to compromise either their HIV treatment or their medical transition.

Body image concerns and eating disorders are prevalent in trans women.^25,75^ Weight gain has been reported with many commonly prescribed antiretroviral medications.^76^ Although there has been no empirical research into the impact of ART-related body shape changes on trans women living with HIV, ART-related lipodystrophy (the abnormal distribution of fat that was associated with older antiretroviral agents) has been shown to cause distress and loss of self-efficacy in cisgender men living with HIV in the United Kingdom.^77^ It is important to explore body image concerns in trans women living with HIV, as this may plausibly impact perceptions of HIV treatment and/or gender transitioning in this population.

There is a dearth of research into the relationship between gender confirmation surgery and HIV risk. In the case of trans women, gender confirmation surgery may involve the surgical construction of a “neovagina” through a variety of procedures, including penile skin inversion, partial bowel transplant, and skin grafting.^78^ It has been hypothesized that trans women with neovaginas may be at an increased risk of HIV acquisition due to changes in the immunological microenvironment as a result of surgery.^79^ Conversely, Poteat et al.^23^ argue that there are some protective factors associated with the neovagina, such as the hardening of tissue after healing. It is likely that other factors moderate the link between gender confirmation surgery and HIV risk, such as the method and quality of surgical construction, the nature and frequency of sexual intercourse, and the use of lubricants. However, it is clear from the existing literature that this remains an under-researched area.

## Access to Health Care

Trans women experience significant barriers to accessing health care, including HIV care.^80–82^ This can be attributed partly to the lack of targeted services, as well as to gender discrimination and transphobic microaggressions perpetrated by health care professionals.^56,74^ Nadal et al.^27^ define microaggressions as subtly antagonistic behaviors, such as misgendering (using incorrect gender pronouns), asking unnecessary intimate questions about gender and sexuality, and wilfully ignoring the existence of transphobia. They also note the adverse impact that microaggressions can have for wellbeing among trans people. It is easy to see how this can adversely affect self-esteem, and how disengagement from health care may in turn constitute an attempt to protect self-esteem.

The perception of poor-quality interactions with health care professionals constitutes a significant barrier to accessing health care among trans patients.^83^ Trans patients may feel that they obtain low-quality health care due to a perceived lack of knowledge of trans people's needs among health care practitioners. This perception may be grounded in reality—only 13% of United Kingdom nurses surveyed felt that they were sufficiently equipped to treat transgender patients.^55^

Stigma can adversely impact engagement with and retention in health care.^84^ An HIV diagnosis can add a further stigmatized identity element to trans gender identity, which can compound oppression and adversely affect the quality of health care.^56,83^ Indeed, the 2016 United Kingdom Stigma Survey found a high prevalence of concerns in trans people about being treated differently by health care providers in a wide range of settings (the overwhelming majority, trans women) with substantial numbers avoiding health care as a result.^29^

Furthermore, lack of family and social support is also associated with disengaging from HIV care among trans women.^85^ Poor engagement with health care services can impact the HIV continuum at all points, from HIV testing to uptake and continued adherence to ART. This in turn may contribute to late diagnosis, increased morbidity and mortality, and an increased risk of onward HIV transmission.

Access to health care may be further compromised by low levels of HIV-related health literacy. Despite the well-documented efficacy of PrEP and post-exposure prophylaxis (PEP), two biomedical HIV prevention approaches,^86,87^ knowledge of these approaches among trans women in the United Kingdom is reported to be low. In their survey of 44 trans people testing for HIV at a sex-on-premises venue in London, Wolton et al.^88^ found that over 70% had no knowledge of PrEP or PEP, and that many expressed concerns about potential drug interactions with hormones. More generally, there is a paucity of data on the acceptability of PrEP and PEP among trans women, which means that potential barriers to uptake of these highly efficacious prevention methods are poorly understood in a group known to be at high risk of HIV acquisition.^89^

Moreover, waiting lists for Gender Identity Clinics, the initial point of contact within health services for trans people wishing to transition medically, are growing.^90^ This can have important implications for the physical and psychological wellbeing of trans people, many of whom face structural violence, and some of whom are living with HIV. For example, Nambiar et al.^91^ report that nearly 40% of trans people attending their specialist sexual health service in the United Kingdom self-medicate with hormones if unable to access hormones in clinical settings and may be unaware of potential drug interactions. However, it is noteworthy that the majority of those reporting nonprescribed hormone replacement therapy in that study were trans men, so there is a need for empirical insight into trans women.

Studies suggest that trans women living with HIV are less likely to adhere to ART and less likely to achieve viral suppression than cisgender patients living with HIV.^92^ A recent case note review of 32 trans women attending a trans sexual health clinic for HIV care found that nearly a third had taken a break from their ART, and that one fifth had a detectable HIV viral load compared with 4% among cisgender patients attending the general HIV service.^88^ It is important to gain an understanding of engagement with HIV care and HIV clinical outcomes in trans women in a larger sample of trans patients in the United Kingdom. It is possible that identity concerns play a significant role in engagement with health care.

## Discussion

Trans women are a key group in the HIV epidemic. This is a group at high risk of HIV acquisition, and those living with HIV are at risk of poorer clinical outcomes as a result of the challenges associated with accessing and engaging with HIV care at all points of the care continuum. In this review, we have discussed both the individual- and macro-level factors that may contribute to these increased risks, highlighting the role of identity process theory and structural violence in providing a theoretical framework that can enhance our understanding of HIV in trans women.

### A model for HIV prevention and care among trans women

**FIG. 2.**
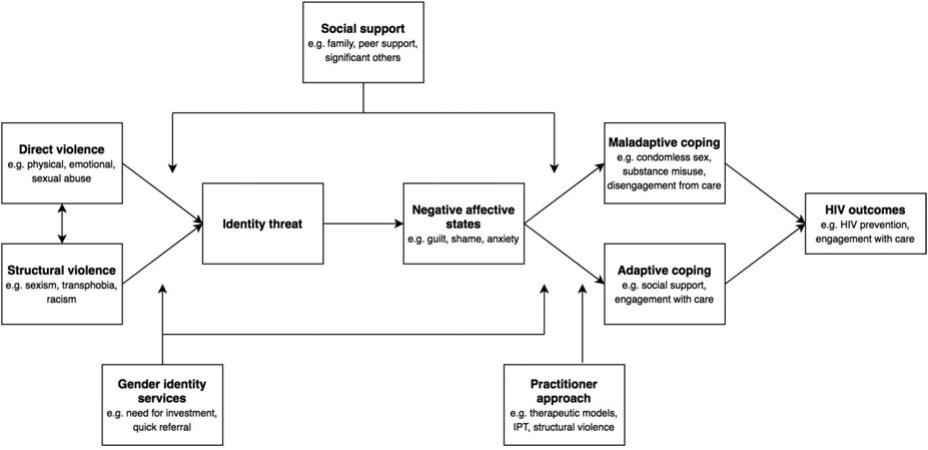
A model for understanding the micro- and macro-level HIV risk factors in trans women (adapted from Jaspal^32^).

In previous research into HIV risk in other populations,^31^ it has been argued that social stressors (one form of structural violence) can threaten identity and give rise to various strategies for coping. This in turn can put the individual at risk of HIV and poor sexual health outcomes. Drawing upon our findings from this narrative review, we offer a modified version of this model as applied to trans women and HIV (Fig. 2).

We hypothesize that both structural violence (e.g., transphobia, sexism, racism) and direct violence (e.g., physical, sexual, and emotional abuse) can threaten identity, but that this is likely to be mediated by the availability of social support and access to affirmative Gender Identity services, which can offer critical support in the face of gender-related adversity. As predicted by identity process theory, identity threat can result in negative affective states (e.g., guilt, shame, and anxiety), which are aversive for psychological wellbeing.

The individual will in turn attempt to cope—either adaptively or maladaptively. Possible maladaptive strategies include condomless sex, substance misuse, and disengagement from care, all of which can increase the risk of HIV and decrease engagement with care among those living with HIV. Adaptive coping strategies include the derivation of social support and engagement with care, which in turn can prevent HIV acquisition and increase engagement with care among those living with HIV.

It is likely that the availability of social support and practitioner engagement mediate the relationship between threat and type of coping response. More specifically, practitioner awareness of identity process theory, structural violence, and therapeutic models can support trans women—both those at risk of, and living with, HIV—in adopting adaptive coping strategies and moving away from those which may be maladaptive.

We suggest that this model be tested in this population through empirical research. We argue that interventions for preventing HIV and promoting better outcomes throughout the HIV care continuum, may benefit from the multilevel tenets of this model. More specifically, it is likely that addressing the multiple and intersecting oppressive forces of transphobia, sexism, and racism (including within health care settings), the provision of greater social support, and a more gender identity affirming approach from practitioners will greatly enhance outcomes in this population. By recognizing the distinctiveness of trans identities and by understanding what factors may promote more positive identities (characterized by a supportive social network), we will be better positioned to support the health and wellbeing of this key population in the HIV epidemic.

### Limitations and next steps

A limitation of this review is that it is a narrative, rather than systematic, review. However, in view of the paucity of literature pertaining to trans women and HIV, and the comprehensive nature of our review, we believe that it constitutes an important contribution to current knowledge. We present a theoretical framework, within which to conceptualize trans women's risk of HIV, and also their engagement with HIV care. It is important to highlight that this is based on our review of current literature and existing theory, and that this model needs to be tested in empirical research. Finally, it is clear that the majority of studies presented in this review were conducted in North America. Findings from these studies may not be applicable in other settings where local HIV epidemiology, the sociodemographic profile of trans women, the legal context, and access to health care may differ. However, there are important and transferable insights to be gained from these studies.

To our knowledge, this is one of the first major reviews of HIV and trans women, which seeks to explicitly incorporate a theoretical framework. Furthermore, in drawing upon identity process theory and the concept of structural violence, we aim to bridge micro- and macro-level processes that may shape HIV risk and impact the HIV care continuum among trans women. A further strength of the review is the inclusion of quantitative (including those using an experimental design) and qualitative studies.

Crucially, this review has enabled us to identify key lacunae in the literature on HIV and trans women in the United Kingdom. First and foremost, it is not appropriate to assume that the needs of trans women are identical to other gender identity groups given the specific oppressive forces that trans women encounter, even within health care settings. Furthermore, it is important to acknowledge that in conflating trans women with MSM, researchers are eliding a fundamental aspect of trans women's identities.

Globally there are notable lacunae in data including: long-term safety and risk of comorbidities among trans women taking hormone therapy; the impact of gender confirmation surgery on HIV infection risk; the role of related syndemics, such as substance misuse (especially chemsex), poor mental health, and violence, in HIV acquisition risk and engagement in HIV care; and qualitative research that sheds light on the lived experience of trans women at risk of, or diagnosed with, HIV. Moreover, existing work often fails to acknowledge the more positive aspects of trans women's lives, and we would therefore encourage researchers to engage with subjects, such as resilience and sexual pleasure, to avoid perpetuating a stereotype of trans women as victims.

The literature on trans women and HIV is nascent in the United Kingdom. This review has highlighted the geographical variation of the HIV epidemic among trans women globally. It is therefore critically important that we generate national surveillance data on the epidemiology and care of trans women living with HIV. Public Health England's recently published data on HIV among trans people in the United Kingdom^24^ is therefore an important first step and we welcome their future work utilizing nonbinary gender categories in their surveillance data collection. We also look forward to results from Positive Voices, a survey of the experiences and health care needs of people attending for HIV care in England, which includes a sample of trans women.^93^

We identified no research into the effectiveness of HIV prevention programs for trans women in the United Kingdom, although there has been a critical review study of interventions in the United States.^94^ Barriers to participating in research among trans women include concerns about stigma, fears about drug interactions (with regard to clinical trials), and previous poor experiences of engaging with health care professionals.^95^ However, future research should aim to address these barriers and acknowledge the needs of this community that can improve HIV prevention efforts, and HIV outcomes.

## Conclusion

In this review, we have drawn upon studies primarily conducted outside of the United Kingdom due to the paucity of United Kingdom-specific data. Much of this work is valuable in enhancing our understanding of HIV risk in trans women, but there is a need for United Kingdom data—both quantitative and qualitative. We believe that there is great scope (and urgency) for research into HIV among trans women, especially in the United Kingdom, to carve out evidence-based pathways for reducing incidence in this group, for addressing inequities in the HIV care continuum, and for improving the health and wellbeing of those living with HIV.

## Abbreviations Used

ART: antiretroviral therapy
MSM: men who have sex with men
PEP: post-exposure prophylaxis
PrEP: pre-exposure prophylaxis
UNAIDS: The Joint United Nations Program on HIV/AIDS
